# Supplementary material for: Effect of Anti-Rheumatic Treatment on the Periodontal Condition of Rheumatoid Arthritis Patients
Source: Int J Environ Res Public Health. 2021 Mar 4;18(5):2529. doi: 10.3390/ijerph18052529 (PMC7967392; doi:10.3390/ijerph18052529)
Supplement: Supplementary file 1 [file ijerph-18-02529-s001.pdf]

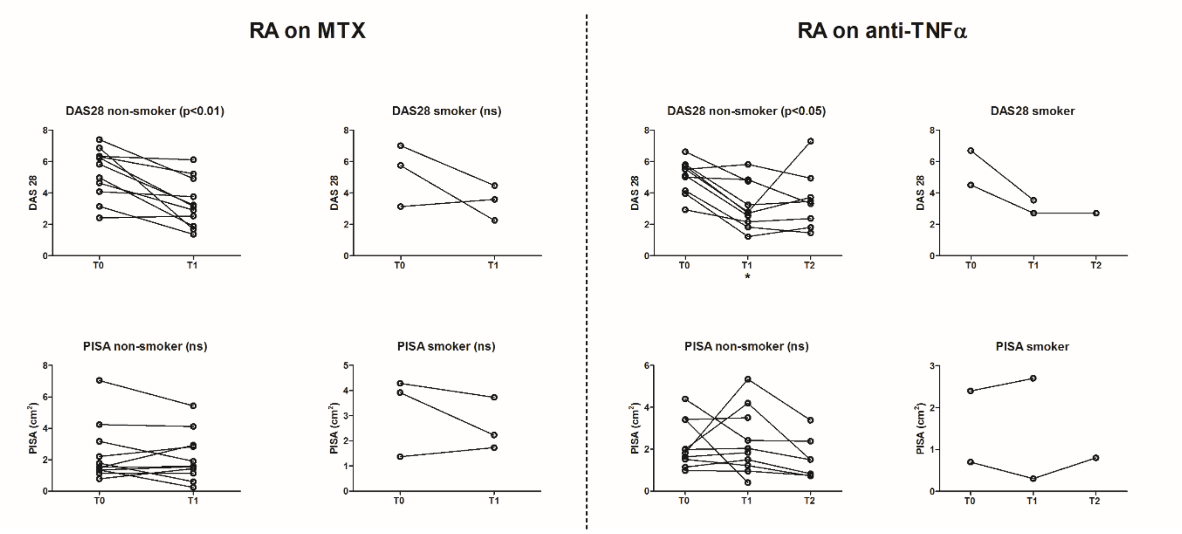

Supplementary Figure S1. Effects of anti-rheumatic treatment (methotrexate (MTX) and anti-tumor necrosis factor-α (anti-TNF in addition to MTX) on disease activity (DAS28) and the periodontal inflamed surface area (PISA).
